# Heparin Attenuates Histone-Mediated Cytotoxicity in Septic Acute Kidney Injury

**DOI:** 10.3389/fmed.2020.586652

**Published:** 2020-12-02

**Authors:** Ziyi Wang, Lijun Wang, Chao Cao, Heng Jin, Yan Zhang, Yancun Liu, Yulei Gao, Xue Liang, Guangping Li, Songtao Shou

**Affiliations:** ^1^Department of Emergency Medicine, Tianjin Medical University General Hospital, Tianjin, China; ^2^Tianjin Key Laboratory of Ionic-Molecular Function of Cardiovascular Disease, Department of Cardiology, Tianjin Institute of Cardiology, The Second Hospital of Tianjin Medical University, Tianjin, China

**Keywords:** heparin, histone, HK-2 cells, apoptosis, septic acute kidney injury

## Abstract

Histones are considered potential risk factors that contribute to the development of septic acute kidney injury (SAKI) by inducing apoptosis and inflammation. This study aimed to explore the protective effects of heparin on septic acute kidney injury through the neutralization of extracellular histones (EH) and to uncover the underlying mechanism. C57BL mice (16 each) were randomly divided into the sham group, the sepsis group (established by cecal ligation and puncture operation, CLP), and the heparin intervention group. Mice in the heparin intervention group received a subcutaneous injection of unfractionated heparin (0.03 IU/g) 4 h after CLP. At 6 h after the operation, nine mice from each group were sacrificed by the removal of the eyeballs to harvest blood samples; the upper half of the right kidney was used as the study sample. Mice renal tubular epithelial cells cultivated in six-well plates were equally divided into five groups. We cultured cells treated with either histone (40 U), histone (40 U) + heparin (25 IU/ml), histone(40U) + lipopolysaccharides (LPS; 10 μg/ml), or histone (40 U) + LPS (10 μg/ml) + heparin (25 IU/ml) for 6 h. For the histone + heparin group and the histone + LPS + heparin group, histone (and LPS) were treated with heparin simultaneously. Mice in the heparin intervention group showed decreased levels of EH4, neutrophil gelatinase-associated lipocalin (NAGL), kidney injury molecule-1 (KIM-1), tumor necrosis factor-α (TNF-α), and interleukin (IL)-6 in the blood serum, longer average 72-h survival rate, significantly decreased kidney tissue edema, and a clearer glomerular structure coupled with decreased protein and mRNA expression levels of kidney apoptosis-related proteins (cleaved Caspase-3/Caspase-3 and Bax/Bcl-2) compared with those in the sepsis group at 6 h after CLP (*P* < 0.05). Meanwhile, cells in the heparin intervention group exhibited lower expression levels of serum EH4 and inflammatory cytokines, a lower apoptosis rate, and decreased expression of apoptosis-related proteins, both at protein and mRNA levels, than those in the histone-stimulated group at 6 h after stimulation (*P* < 0.05). Heparin may alleviate apoptosis and inflammation through the neutralization of histones, thus playing a protective role against septic acute kidney injury.

## Introduction

Extracellular histones (EH) have been shown to exhibit cytotoxicity that can induce apoptosis in HK-2 cells *in vitro* (1). Exposure to antibody directed at histone H4 in cells pretreated with H4 has been shown to effectively alleviate the damaging effects of histones on cell integrity and inflammation (2). Zarjou et al. have reported that EH can lead to a series of acute kidney injury (AKI) manifestations *in vivo*, such as endothelial cell activation, increased vascular permeability, and leukocyte recruitment (3). Using a murine model of septic AKI (SAKI), animals receiving anti-histone antibody exhibited much less injury than those without treatment (4), indicating that histones may be potential targets for SAKI, although the underlying mechanisms involved in SAKI are not entirely clear. Apoptosis, especially apoptosis of HK-2 cells, has drawn much research attention. It has been assumed that the increased production of reactive oxygen species (ROS) may activate cell apoptosis (5).

Heparin is known to carry the highest negative charge among all biomolecules known. Accordingly, histones have a stronger binding affinity for heparin than for DNA molecules (6). Thus, heparin may attenuate EH-mediated cytotoxicity through the neutralization of opposite charges, both in HK-2 cells and in a SAKI mice model. However, limited research attention has been given to the protective effect of heparin in neutralizing histones that induce cytotoxicity. In this study, the roles histones and heparin play in apoptosis and inflammation were analyzed using *in vivo* and *in vitro* approaches. In addition, the relationship between histones and ROS in cell apoptosis was evaluated using HK-2 cells.

## Methods

### Reagents

EH was purchased from Roche (China). Heparin was purchased from Tianjin Biochemical Pharmaceutical Company. Anti-Bcl-2 antibody, anti-Bax antibody, anti-Caspase-3 antibodies, horseradish peroxidase (HRP)-labeled goat anti-mouse IgG antibody, and HRP-labeled goat anti-rabbit IgG antibody were purchased from Abcam (USA). Cleaved Caspase-3 antibody was purchased from CST/Cyxnet (USA). Mouse tumor necrosis factor (TNF)-α ELISA kit and TransStart Top Green qPCR Supermix were purchased from Beijing TransGen Biotech. The mouse histone H4 ELISA kit, mouse NAGL ELISA kit, and mouse KIM-1 ELISA kits were purchased from Shanghai Guangrui Biotech. Purified anti-β-actin and the fluorescein isothiocyanate (FITC)–annexin V apoptosis detection kit with 7-amino-actinomycin D (7-AAD) were purchased from Biolegend (USA). Fetal bovine serum was purchased from GIBCO (USA). DCFH-DA probe was purchased from Shanghai Beyotime Biotechnology.

### Murine Model

Adult male C57BL/6J mice, aged 6–8 weeks and weighing 19–21 g, were purchased from Peking University Experimental Animals Center (Beijing, China), housed in a pathogen-free environment in the animal center of Tianjin Medical University, and allowed to acclimate to their surroundings for 1 week. Standard mice chow and water were available to the animals during the course of the experiment *ad libitum*. The mice were equally divided into three groups (*n* = 12 mice/group): sham, sepsis, and heparin intervention groups. The surgical procedure was performed as follows: male mice were fasted for at least 12 h and then anesthetized by intraperitoneal injection of tribromoethanol (10 mg/kg). For the sepsis and the heparin intervention groups, the cecum was exposed after mid-line laparotomy and ligated immediately below the ileo-cecal valve without causing intestinal obstruction. After being punctured twice with an 18G needle, the cecum was placed back in the peritoneal cavity, and the abdominal wall was closed in two layers. Then, the heparin intervention group was also administered with heparin (3 mg/kg) by intraperitoneal injection at 4 h after the CLP operation. For the sham group, the cecum was exposed, and then the abdominal wall was closed in two layers. All the three groups were treated with normal saline just after operation to mimic clinical therapy. At 6 h after the operation, nine mice from each group were sacrificed by the removal of eyeballs to harvest the blood, after which the kidneys were removed immediately, and the upper pole of the right kidney was used as the study sample. The levels of kidney injury factors (NAGL and KIM-1) in the mice serum and kidneys were evaluated separately by ELISA and PCR. We also administered calf thymus histone (10 mg/kg) by intraperitoneal injection into the mice (*n* = 3), and the survival of these four groups at 72 h was recorded. Our experiments were carried out in strict accordance with the international ethical guidelines and National Institutes of Health Guide concerning the care and use of laboratory animals, with protocol being approved by the Institutional Animal Care and Utilization Committee of Tianjin Medical University.

### Cell Culture

HK-2 cells were obtained from Tianjin Institute of Urology and were cultured in an incubator (SANYO, Japan) at Tianjin Institute of Cardiology under standard conditions (37°C, 5% CO_2_); the experiments were carried out after two passages. The cells were divided into three groups: control, histone, and heparin intervention groups. Each group contained an approximately equal number of cells. Cells in the control group received no intervention, whereas cells in the histone group were treated with EH (H4). Meanwhile, cells in the heparin intervention group were treated with heparin in the presence of histone. In order to explore the effects of lipopolysaccharides (LPS) on the apoptosis of HK-2 cells, three additional treatment groups were used: LPS, LPS + histone (with LPS and histone added simultaneously), and LPS + histone + heparin (with LPS, histone, and heparin added simultaneously) groups.

### ELISA Measurement

The levels of H4, TNF-α, IL-6, NAGL, and KIM-1 from supernatants or blood serum were measured by ELISA kits, according to the manufacturer's protocols. Plates were read in a microplate reader at OD 450. All the samples were tested in triplicate.

### Flow Cytometry Analysis

Cell apoptosis was tested by flow cytometry using an annexin V–FITC apoptosis detection kit. HK-2 cells were washed twice with phosphate-buffered saline (PBS) and resuspended in 100 μl of 1× binding buffer mixed with 5 μl of annexin-V–FITC and 2.5 μl of 7-AAD staining solution for 15 min in the dark at room temperature. After 15 min of incubation, an additional 400 μl of binding buffer was added, and then the cells were analyzed using a flow cytometer (BD, USA). The production of ROS was tested by flow cytometry using an DCFH-DA probe. HK-2 cells were washed twice with PBS and resuspended in 100 μl PBS mixed with 10 μM DCFH-DA for 30 min in the dark at room temperature. Then, the cells were washed with PBS three times, and 300 μl PBS was added before analysis by flow cytometry. 7-AAD and annexin-V assay Q2 + Q3 were used to perform the apoptosis rate (7).

### PCR Evaluation of Gene Expression

Trizol reagent (Life Technologies, USA) was used to extract total RNA from cells. Subsequently, cDNA was synthesized by FastQuant RT Kit (TianGen Biotech, Beijing), using the GeneAmp PCR System 2400 (Perkin Elmer, USA). SYBR Green Master Mix was used to amplify gene targets by real-time fluorescence quantitative PCR (qPCR) in accordance with the manufacturer's protocol and the 7500 Fast Real-Time PCR System (ABI, USA) to perform qPCR analysis. The specific primer sequences used for gene amplification are shown in Table 1. The cycling conditions were as follows: 95°C for 30 s, 60°C for 30 s, and 72°C for 30 s. The expression levels of targeting mRNAs were normalized to that of housekeeping gene GAPDH and expressed as a fold of control.

**Table 1:** Primer sequences used in RT-PCR.

	**Forward primer**	**Reverse primer**
GAPDH	TGCACCACCAACTGCTTAG	GATGCAGGGATGATGTTC
Bax	AGCTGCAGAGGATGATTGCT	ATGGTTCTGATCAGCTCGGG
Bcl-2	CCACCTGTGGTCCATCTGA	GACTGGACATCTCTGCGAA
Caspase-3	TGCTCACGAAAGAACTGTACT	GACAGCTTTCTCATTTGGCATA

### Western Blotting

Apoptosis-mediated proteins were analyzed by western blotting. The total protein was extracted by radio-immunoprecipitation assay and phenylmethane sulfonylfluoride following the standard protocols for extracting protein. The protein concentrations were quantified using the BCA Protein Assay kit. Samples were electrophoresed in 10 or 8% SDS-PAGE gel and transferred onto a polyvinylidene fluoride membrane. Then, the membrane was blocked in 5% dried milk at 4°C overnight. The membrane was washed with Tris-buffered saline Tween-20 (TBST) three times, followed by incubation with a secondary antibody at room temperature for 40 min. After washing with TBST again, to observe protein signals, substrate luminol reagent and HRP substrate solution were added onto the membrane, 1 ml/membrane, and membrane signals were revealed by an enhanced chemiluminescence immunoblot detection system. The staining intensity of the bands was quantitated by densitometry through ImageJ software. The antibodies used in our study are described above in the “Reagents.” Protein expression levels were defined as gray value, standardized to the housekeeping gene β-actin, and expressed as a fold of control. All experiments were performed in triplicate and three times independently.

### Hematoxylin and Eosin Staining Measurement

To observe the effect of heparin on organ histomorphology in CLP-induced septic mice, kidneys were inflated and fixed with 4% methanol-free formaldehyde for 48 h and embedded in paraffin. Tissue sections of 5-mm thickness were cut and stained with hematoxylin and eosin (H&E) following the standard staining protocols for histological analysis. Tubular injury scores were assigned to H&E-stained kidney sections by an experienced kidney pathologist who was blinded to the identity of the samples. We adopted published criteria as described for each parameter (8). Tubular injury degeneration was defined as including vacuolization, luminal cell casts, and acellular/atrophic changes, and scoring was as follows: 0 = none detected, 1 = 1–10% tubules involved, 2 = 10–25% tubules involved, 3 = 25–50% tubules involved, and 4 = >50% tubules involved. Tubulointerstitial inflammation was defined as the presence of lymphocytes in perivascular and interstitial cortical areas, and scoring was as follows: 0 = no significant inflammation, 1 = 1–10% foci in perivascular areas, 2 = 10–25% of cortex involved, 3 = 25–50% of cortex involved, and 4 = >50% of cortex involved.

### Statistical Analysis

SPSS (version 22.0) was used to conduct all statistical analysis. Statistical analysis was accomplished based on the data from three independent experiments. Data are presented as mean ± standard deviation (SD). Statistical differences between the means of multiple groups were analyzed by one-way analysis of variance. *P* < 0.05 was considered to indicate a statistically significant difference.

## Results

### Heparin Administration Can Improve the Survival Rate of SAKI Mice Induced by CLP

**Figure 1:**
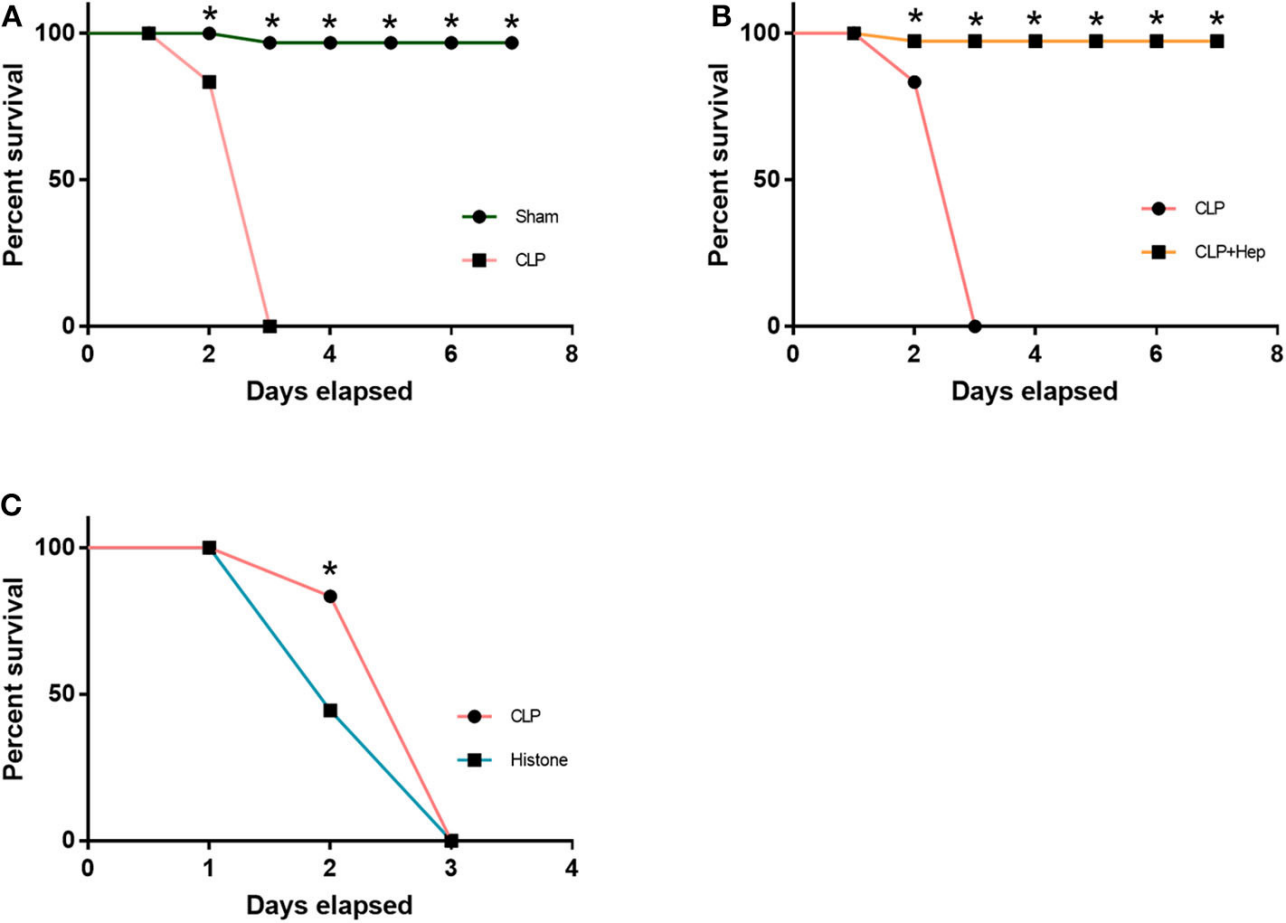
Heparin improves the survival rate. The 72-h survival rates of the sham group, cecal ligation and puncture operation (CLP) group, CLP + heparin group, and histone group (instead of CLP, received 10 μg/ml histone intraperitoneally) were observed. **(A,C)** The CLP group and the histone injection group had a lower survival rate than the Sham group. **(B)** The heparin intervention group dramatically improve the 72-h survival rate. Data are presented as mean ± SD, *n* = 7 per group of the representative data from three independent experiments; *P < 0.05.

Mice were divided into four groups: sham group, sepsis group, heparin intervention group, and histone group. The heparin intervention group was also administered with heparin (3 mg/kg) by intraperitoneal injection at 4 h after the CLP operation. The histone group was administered with calf thymus histone (10 mg/kg) by intraperitoneal injection. The 72-h survival rate of the four groups was monitored in our study. As illustrated in Figures 1A,C, while all sham-operated mice survived to the end of the observation period (except one that died on day 2), all mice in the sepsis group died within 3 days. As illustrated in Figure 1B, the survival rate of mice in the sepsis group and the histone injection group were lower than that in the sham group. The survival rate of mice in the heparin intervention group was essentially improved compared with that in the sepsis group.

### Heparin Can Ameliorate the Abnormal Morphology Observed in the Kidneys of SAKI Mice

**Figure 2:**
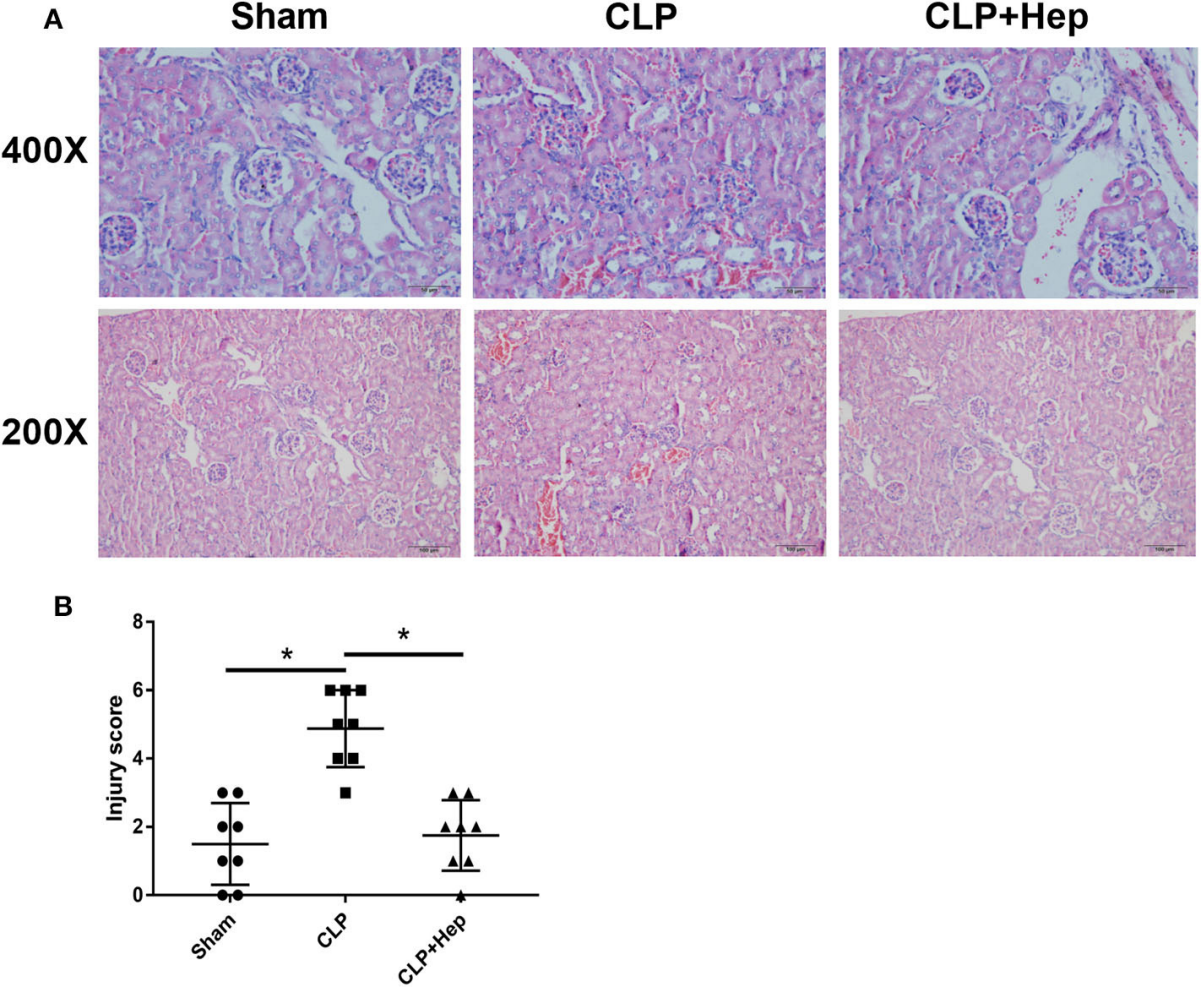
Heparin ameliorates the histology of kidney in a septic mice model induced by cecal ligation and puncture operation. **(A)** Kidney histology changes of the sham group, sepsis group, and heparin intervention group were examined by hematoxylin and eosin (H&E) staining. Representative images of H&E-stained kidneys of the three groups at low (lower panels) and high (upper panels) magnification; scale bars are shown in figures. **(B)** Tubular injury scores were assigned to H&E-stained kidney sections to show kidney histology changes (*P < 0.05). The histopathological findings shown are representative of a similar histology observed in more than three mice examined for each group.

The kidney structure changes were shown by H&E staining. As illustrated in Figure 2, mice in the sepsis group presented anomalies in kidney structure. However, mice in the heparin intervention group had significantly improved anomalies. Renal tubular injury, quantified based on the tubular injury scores of H&E-stained kidney sections, was also significantly lower in the heparin intervention group, indicating that heparin is sufficient to protect the kidneys from septic damage.

### Heparin Can Decrease Levels of NAGL and KIM-1 in SAKI Mice Induced by CLP

**Figure 3:**
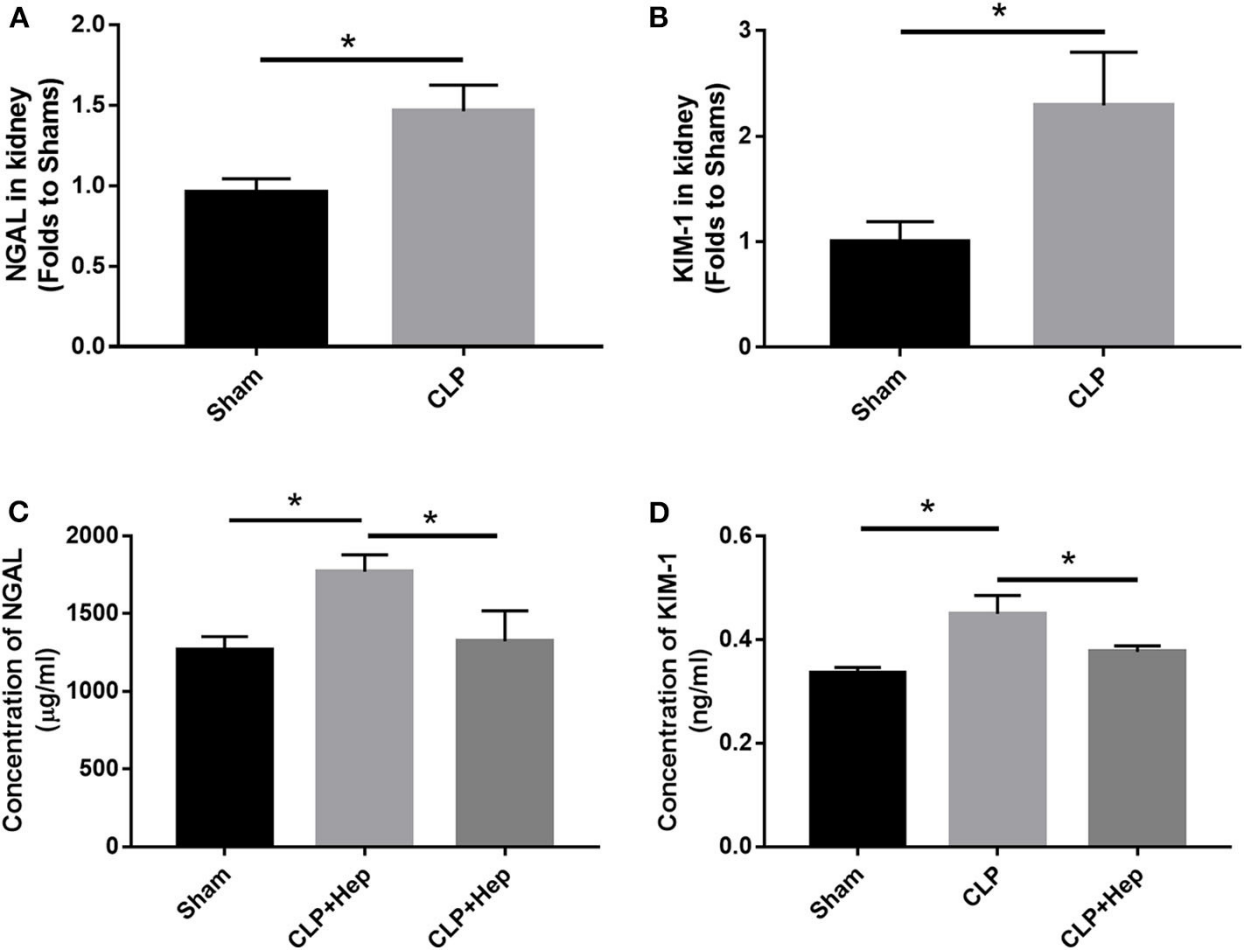
Heparin decreases the expressions of NGALF and KIM-1 *in vivo*. **(A,B)** PCR analysis showing the mRNA expression levels of NGALF and KIM-1 in the kidney in each mouse group. **(C,D)** ELISA revealed the protein expression level of circulating NGALF and KIM-1 in the blood serum in each mouse group. Data are presented as mean ± SD, *n* = 3 per group of the representative data from three independent experiments; *P < 0.05.

The *in vivo* expression levels of NAGL and KIM-1 in the kidneys of mice were determined using ELISA and PCR. Our data showed that, at 6 h after CLP, the levels of NAGL and KIM-1 were significantly higher in the sepsis group than in the sham group and that heparin could dramatically decrease the protein expression levels of both NAGL and KIM-1 (Figure 3).

### Heparin Can Decrease H4 Levels and Pro-inflammatory Cytokine Release in HK-2 Cells Treated With Histone

**Figure 4:**
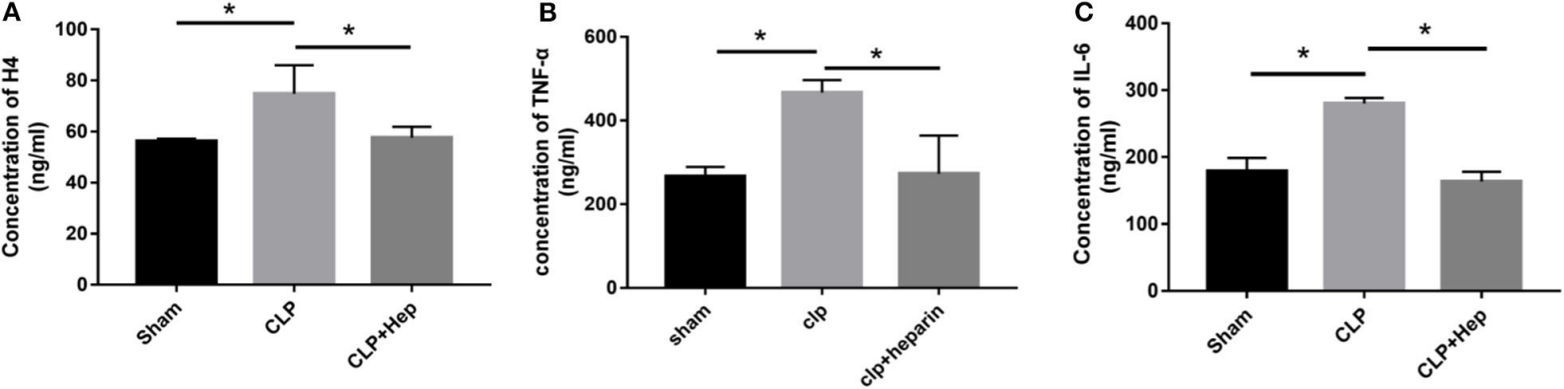
Heparin decreases the expressions of inflammation-related factors and level of EH4 *in vivo*. At 6 h before the onset of surgery, blood samples acquired by removing the mice's eyeballs were used to determine the expression levels of H4 **(A)**, TNF-α **(B)**, and IL-6 **(C)** in in blood serum by ELISA. Data are presented as mean ± SD, *n* = 3 per group of the representative data from three independent experiments; *P < 0.05.

ELISA was performed to evaluate the EH levels in the blood serum of mice in all three groups. Our results showed that mice in the sepsis group exhibited higher expression levels of H4 in serum (Figure 4), indicating that the levels of EH increased during the development of SAKI. Administration of heparin effectively decreased the expression of H4.

Cytokines are known to play important roles in the regulation of the immune response and inflammation. IL-6 and TNF-α are the main pro-inflammatory factors that regulate early responses. TNF-α is the most well-studied pro-inflammatory cytokine and has been shown to be involved in sepsis. Here we evaluated the *in vivo* release of inflammatory cytokines by ELISA. The levels of TNF-α and IL-6 in the serum from mice in the sepsis group (and in the cultured cells of the histone group for *in vitro* analysis) were higher (Figure 4) compared with those in the sham group, and heparin effectively decreased the levels of TNF- α and IL-6 in the samples of the heparin intervention group.

### Heparin Can Regulate Apoptotic Proteins in HK-2 Cells Treated With Histone

**Figure 5:**
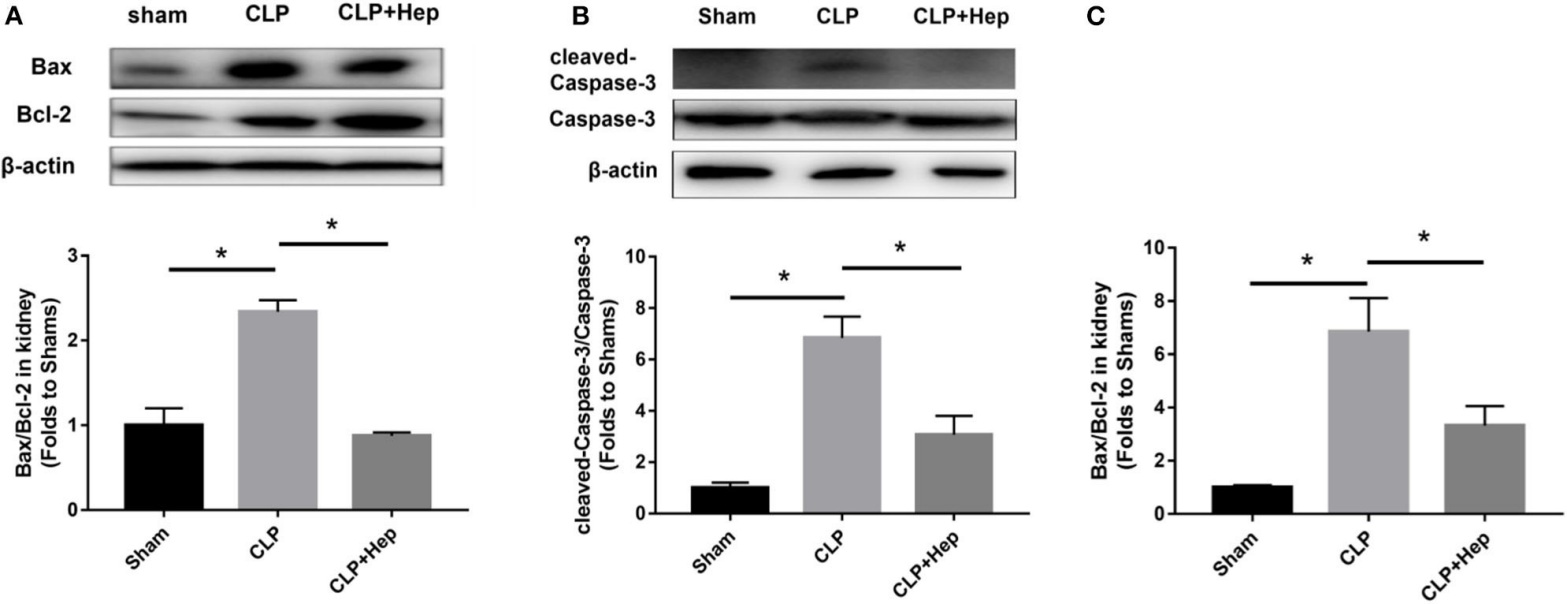
Heparin decreases the expression levels of apoptosis-mediated proteins *in vivo*. **(A,B)** The protein expression of Bax/Bcl-2 and cleaved Caspase-3/Caspase-3 in HK-2 was analyzed by western blotting and quantified by densitometry. The mice were subjected to cecal ligation and puncture operation (CLP) surgery. At 2 h before the onset of CLP, the mice were treated with heparin. The levels of protein in the kidney were determined by western blotting and quantified by densitometry. **(C)** The mRNA expression of Bax/Bcl-2 was analyzed by SYBR Green qPCR. The ratio of Bax/Bcl-2 in the sepsis group was higher than that in the sham group. The ratio of Bax/Bcl-2 in the heparin intervention group was lower than that in the sepsis group. Data are presented as mean ± SD, *n* = 3 per group of the representative data from three independent experiments; *P < 0.05.

Apoptotic proteins, including Caspase-3, cleaved Caspase-3, Bax, and Bcl-2, have been reported to be involved in apoptotic pathways. We evaluated the levels of apoptotic proteins *in vivo* by western blot analysis. As shown in Figure 5, the ratio of cleaved Caspase-3/Caspase-3 and Bax/Bcl-2 expression was increased in mice of the sepsis group, and heparin dramatically reduced the levels of apoptotic proteins. Consistent with the data obtained from western blotting analysis, our PCR results also showed that, although the ratio of Bax/Bcl-2 gene expression was dramatically increased in mice of the sepsis group, the ratio decreased in samples from the heparin intervention group.

### Pre-study Was Performed to Determine the Time Point and the Concentration in HK-2 Cells Treated With Histone

**Figure 6:**
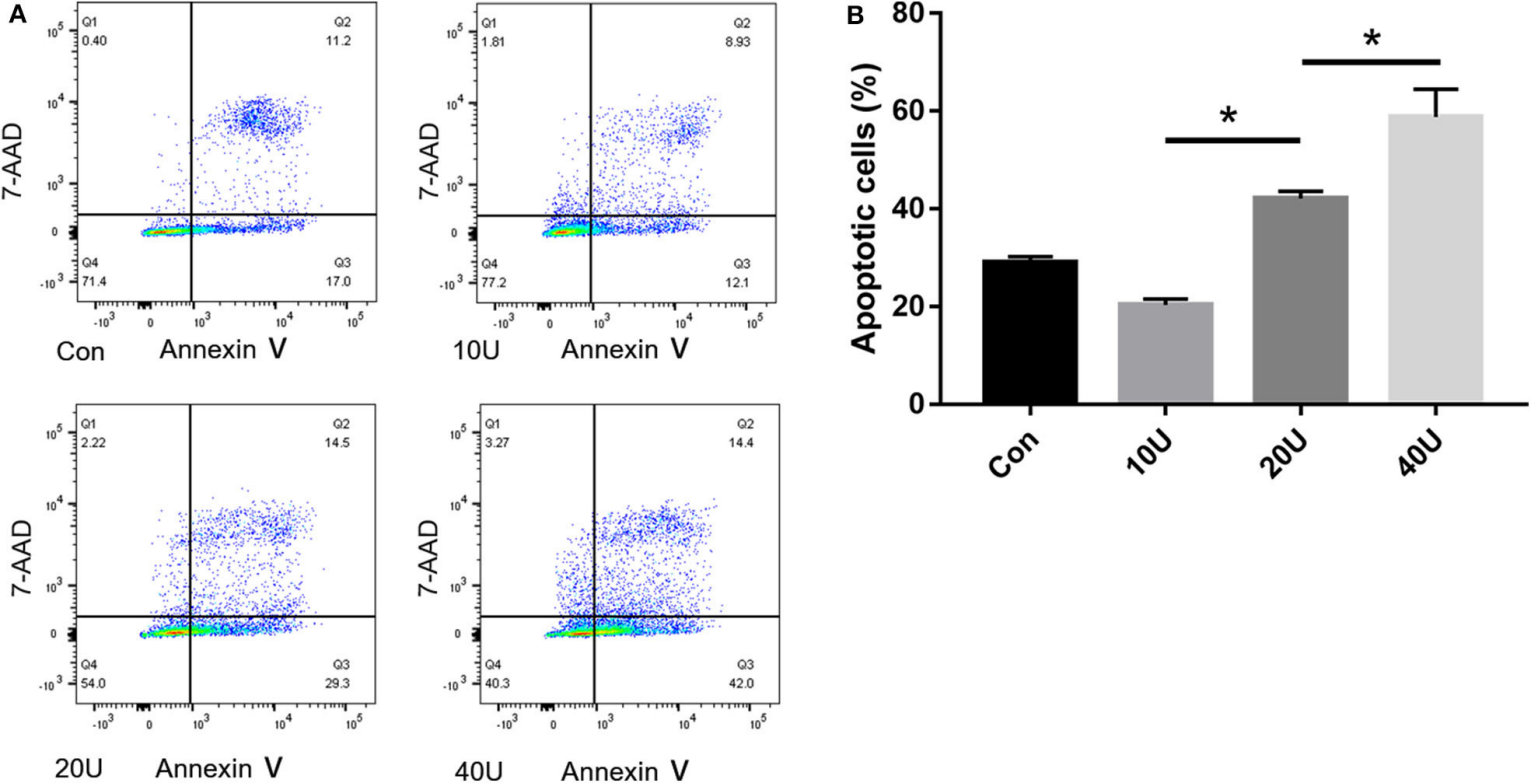
Pre-study to find an efficient concentration of histone. **(A)** HK-2 cells were treated with histones (10 U/20 U/40 U) at 6 h. Flow cytometry was used to evaluate apoptosis rate. **(B)** The apoptosis rate of 20 U and 40 U are higher in comparison to the control. The apoptosis rate of 40 U group is higher than that in 20 U group. There is no significant difference between 10 U group and the control group (*P* < 0.05). Data are presented as mean ± SD (*n* = 3 per group) of the representative data from three independent experiments; *P < 0.05.

**Figure 7:**
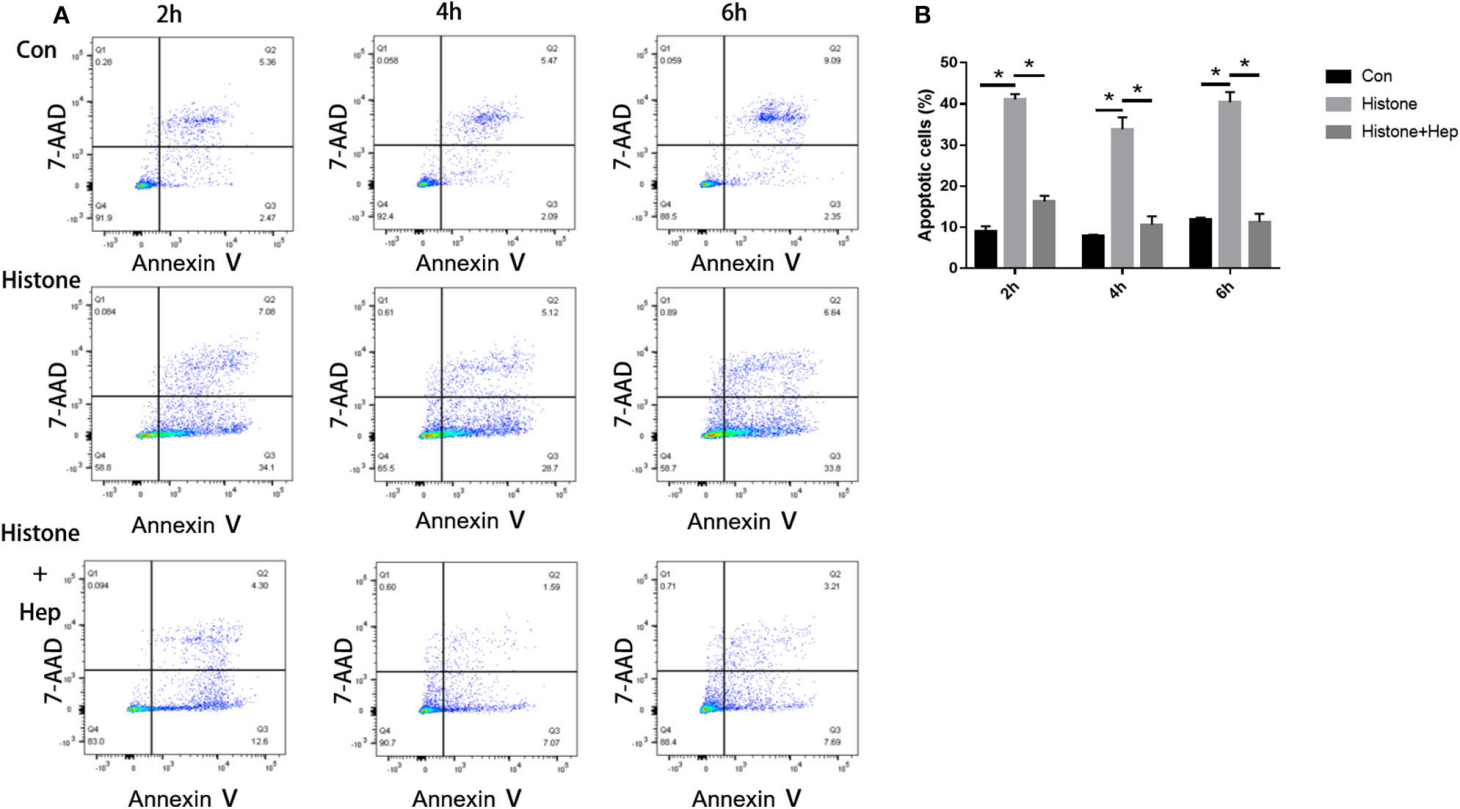
Pre-study to find an efficient time point. **(A)** Cells were treated with 40 U histone cultured for 2, 4, and 6 h. Flow cytometry was used to evaluate apoptosis rate **(B)** Data showed that heparin could significantly reduce the apoptosis rate in each group (*P* < 0.05). Data are presented as mean ± SD, *n* = 3 per group of the representative data from three independent experiments; *P < 0.05.

**Figure 8:**
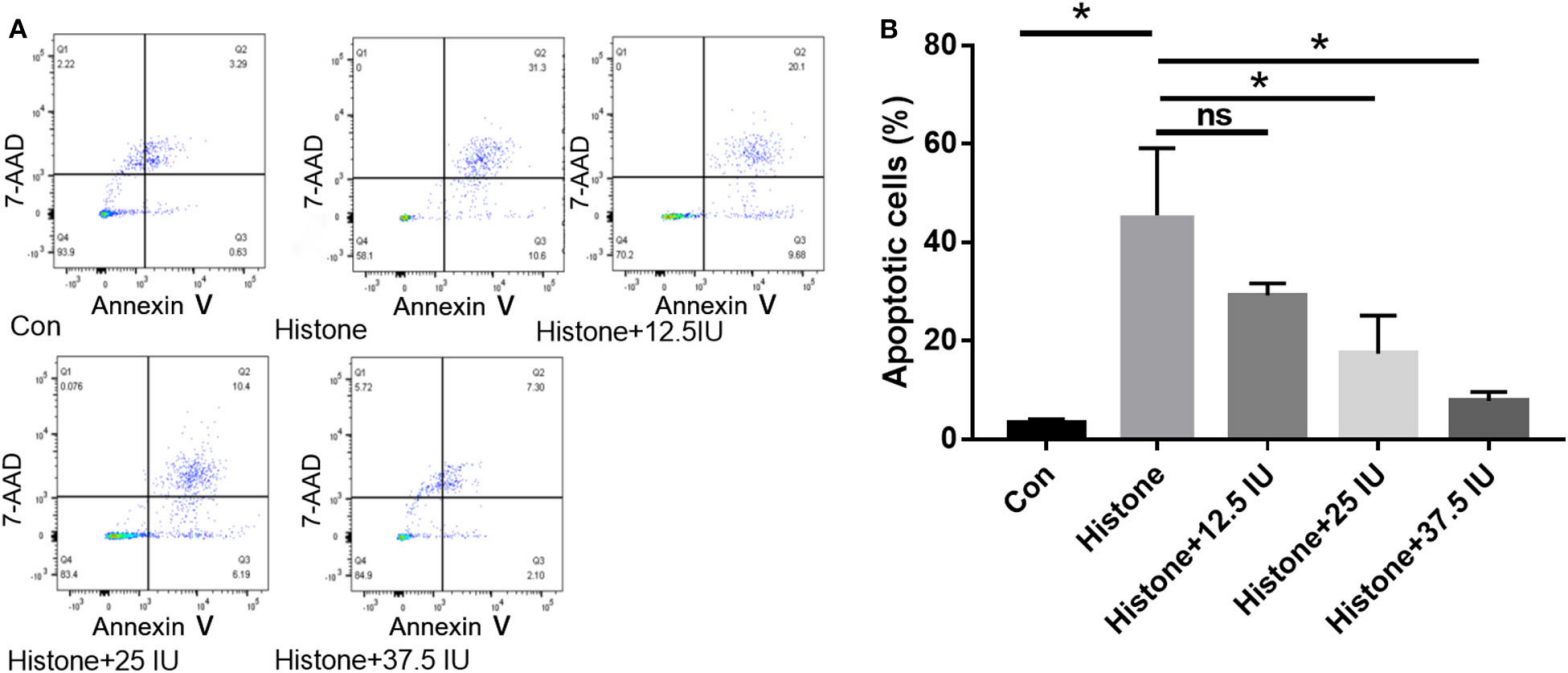
Pre-study to find an efficient concentration of heparin. **(A)** HK-2 cells treated with 40 U histone were administered with different concentrations of heparin and cultured for 6 h. Flow cytometry was used to evaluate apoptosis rate. **(B)** The proportion of apoptotic cells was significantly lower at doses of 25 and 37.5 IU/ml in comparison to 12.5 IU/ml (*P* < 0.05). Data are presented as mean ± SD, *n* = 3 per group of the representative data from three independent experiments; *P < 0.05.

In our pre-study, as shown in Figure 6, HK-2 cells were treated with histones at different concentrations for 6 h. Our data showed that the proportion of apoptotic cells (Q2 + Q3) in the control group was only 29.05 ± 1.22%; the proportion of apoptotic cells (Q2 + Q3), following treatment with 10 U, 20 U, and 40 U of histone, was determined to be 20.40 ± 1.67, 42.13 ± 1.47, and 58.8 ± 5.69%, respectively. The increase in apoptotic cells following treatment at a higher concentration of histone (40 U) was more pronounced than in cells exposed to a moderate amount of histone (20 U), so we chose 40 U as an efficient concentration for histone. As shown in Figure 7, HK-2 cells were treated with 40 U histone and cultured for 2, 4, and 6 h before analysis by flow cytometry. The proportion of apoptotic cells (Q2 + Q3) increased in cells treated with histone from each time point compared to the same time points in the control group without histone treatment, and heparin could significantly alleviate apoptosis in cells treated with histone from each time point. For the convenience of the study, we chose 6 h as an efficient time point. As shown in Figure 8, HK-2 cells treated with 40 U histone were cultured with heparin at different concentrations for 6 h before the flow cytometry analysis. The proportion of apoptotic cells (Q2 + Q3) was significantly lower following exposure to heparin at concentrations of 25 and 37.5 IU/ml, compared to apoptotic cells observed following the 12.5 IU/ml treatment (*P* < 0.05), so we chose 25 IU/ml as an efficient concentration for heparin.

### Heparin Can Decrease EH4 Levels and Pro-inflammatory Cytokine Release in HK-2 Cells Treated With Histone

**Figure 9:**
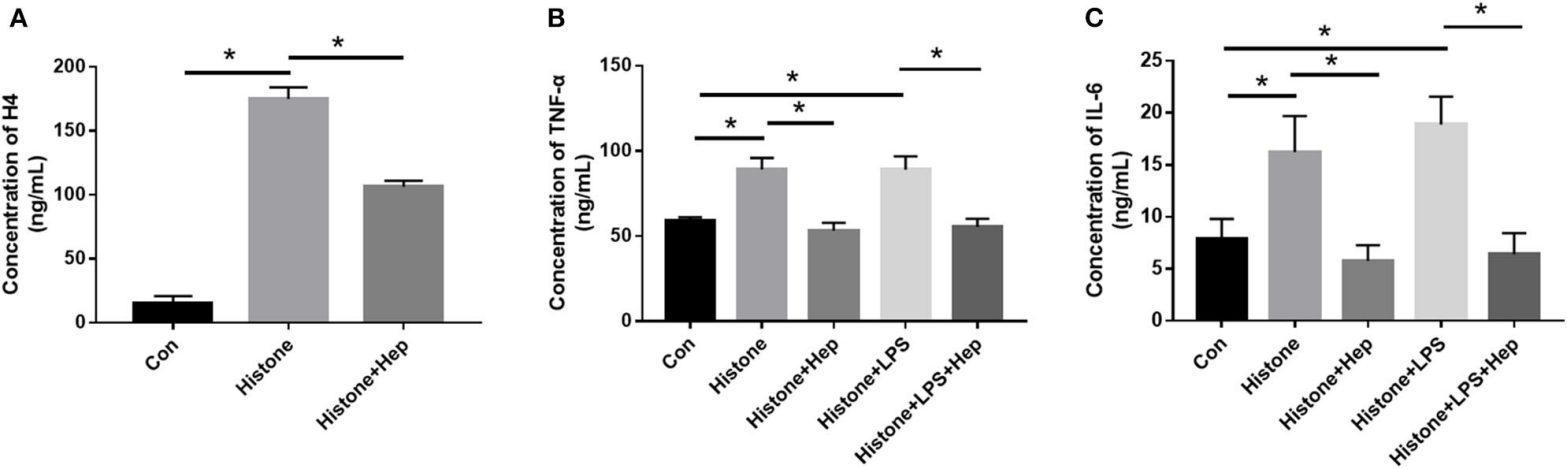
Heparin decreases the expression of inflammation-related factors and level of EH4 in HK-2 cells treated with histone. At 6 h after cultivation, the expression levels of H4 **(A)**, TNF-α **(B)**, and IL-6 **(C)** in cell supernatants were determined by ELISA. Data are presented as mean ± SD (*n* = 3 per group of) the representative data from three independent experiments; *P < 0.05.

HK-2 cells were divided into five groups. Besides the control (Con) group, we cultured HK-2 cells treated with either histone (40 U), histone (40 U) + heparin (25 IU/ml), histone (40 U) + LPS (10 μg/ml), or histone (40 U) + LPS (10 μg/ml) + heparin (25 IU/ml) for 6 h. For the histone + heparin group and the histone + LPS + heparin group, histone (and LPS) was treated with heparin simultaneously. At 6 h later, the levels of EH4, IL-6, and TNF-α present in the cell culture supernatant of all the five groups were evaluated. The ELISA data showed that the cell culture supernatant of the histone (and LPS) stimulation group had significantly higher levels of EH4, IL-6, and TNF-α than those present in the control group (Figure 9). The ELISA data also showed that the cell culture supernatant of the heparin intervention group had significantly lower levels of EH4, IL-6, and TNF-α than those present in the histone (and LPS) stimulation group (Figure 9). Our results clearly demonstrated that heparin could decrease the levels of histone and pro-inflammation factors in the culture supernatant of HK-2 cells pretreated with histone (and LPS).

### Heparin Can Inhibit Apoptosis Induced by Histone Along With LPS in HK-2 Cells

**Figure 10:**
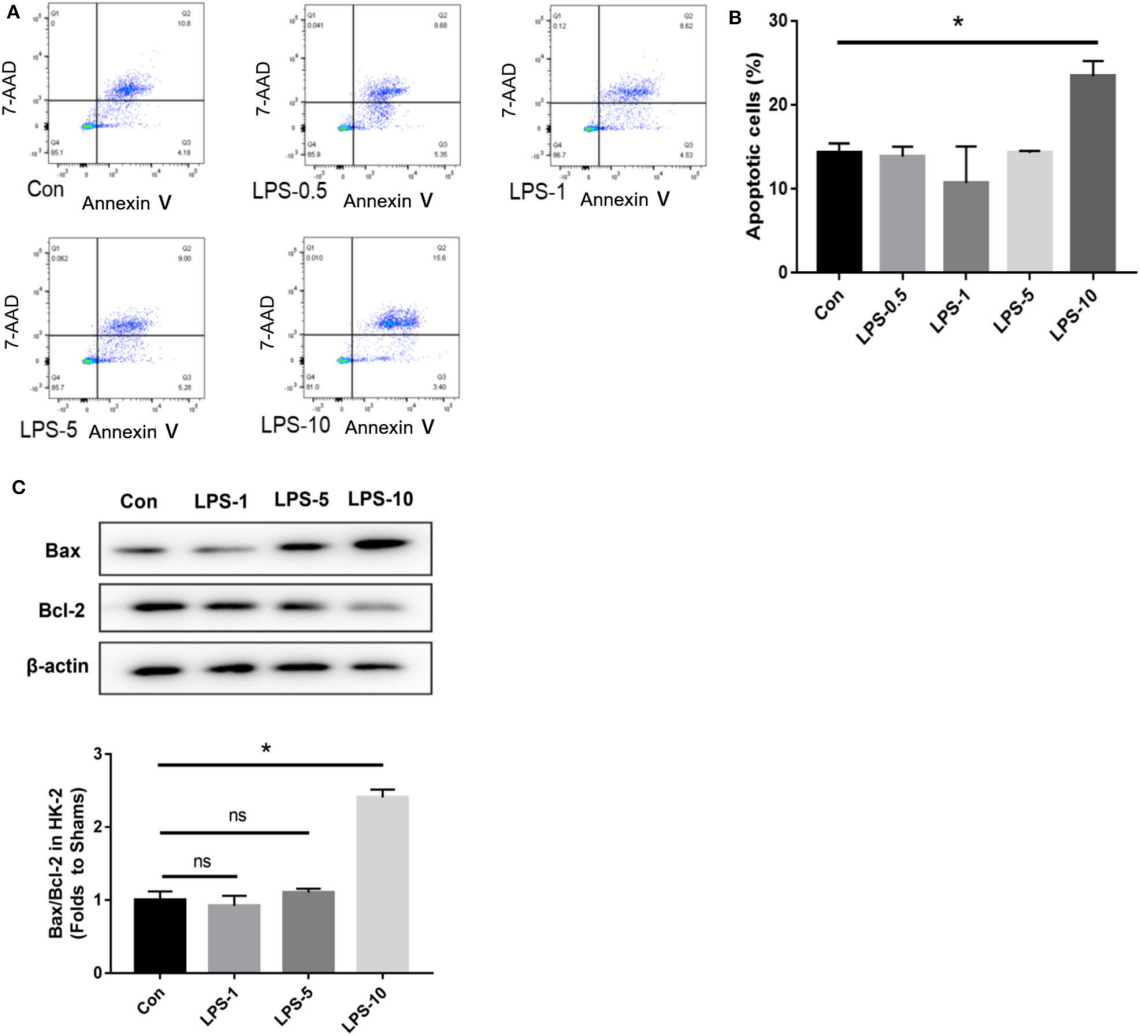
The cytotoxic effect of lipopolysaccharides (LPS) on HK-2 cells. The HK-2 cells were treated with LPS at doses of 0.5, 1, 5”, and 10 μg/ml. **(A,B)** A flow cytometry analysis showed that only the only 10 μg/ml group increased the proportion of apoptotic cells (Q2 + Q3) in comparison to the control group. **(C)** HK-2 cells were analyzed by western blotting and quantified by densitometry. The ratio of Bax/Bcl-2 was higher only at the dose of 10 μg/ml compared with the control group. Data are presented as mean ± SD (*n* = 3 per group) of the representative data from three independent experiments; *P < 0.05.

**Figure 11:**
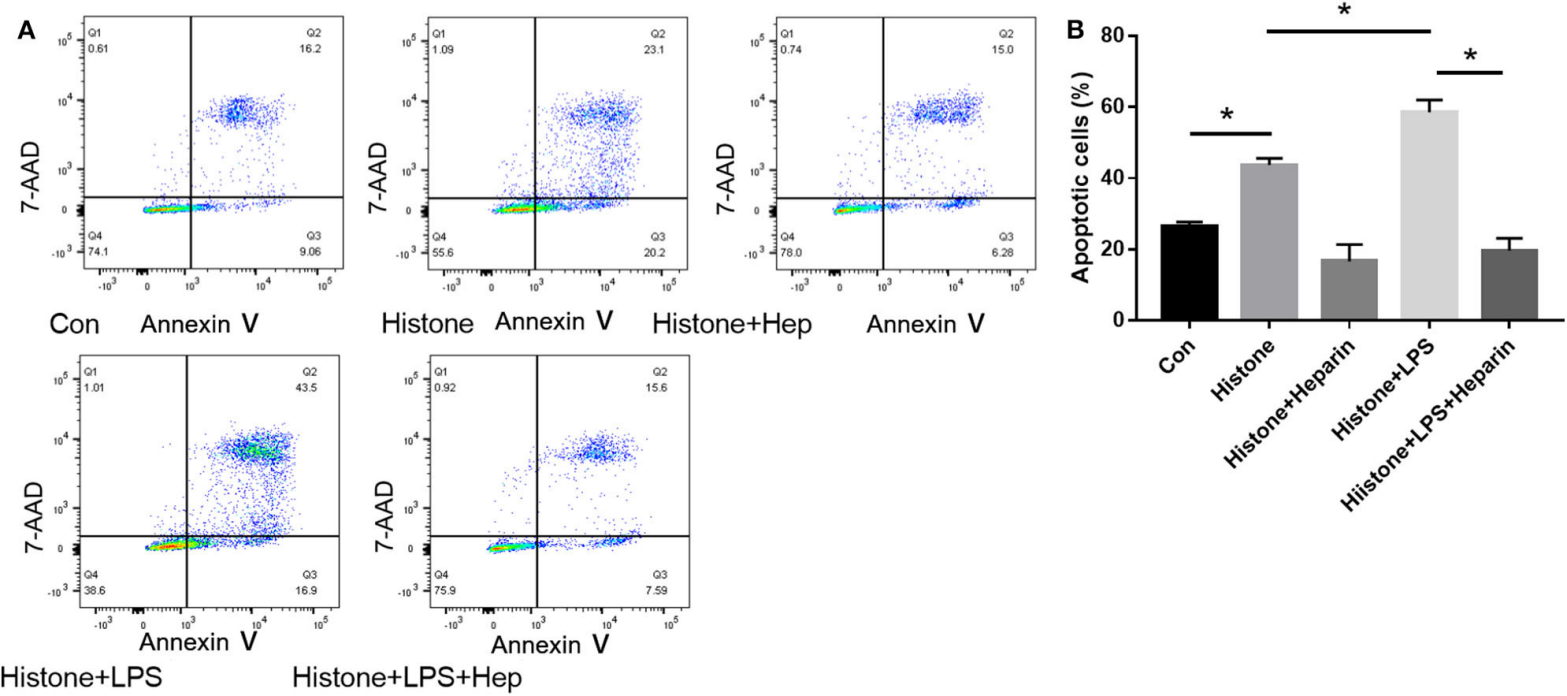
Histone, along with lipopolysaccharides (LPS), increases the apoptosis rate and heparin alleviates it. **(A)** We equally divided cells into the control (con) group, histone (40 U) group, histone (40 U) + LPS (10 μg/ml) group, histone (40 U) + heparin (25 IU/ml) group, and histone (40 U) + LPS (10 μg/ml) + heparin (25 IU/ml) groups and evaluated the proportion of apoptotic cells (Q2 + Q3) in each group. **(B)** For histone and histone + LPS groups, the proportion of apoptotic cells (Q2 + Q3) increased significantly (*P* < 0.05) in comparison to the control group. Heparin remarkably reduced the cell apoptosis rate both in histone and histone + LPS groups (*P* < 0.05). Data are presented as mean ± SD, *n* = 3 per group of the representative data from three independent experiments; *P < 0.05.

In our pre-study, as shown in Figure 10, HK-2 cells were treated with LPS at an increasing concentration of 0.5, 1, 5, and 10 μg/ml. Only cells treated with 10 μg/ml LPS had an increased proportion of apoptotic cells (Q2 + Q3) compared to the control group (*P* < 0.05). In parallel, western blotting showed that only cells treated with 10 μg/ml LPS had an increased ratio of Bax/Bcl-2 compared to the control group (*P* < 0.05). We further cultured HK-2 cells treated with either histone (40 U), histone (40 U) and heparin (25 IU/ml), histone (40 U) and LPS (10 μg/ml), or histone (40 U) + LPS (10 μg/ml) + heparin (25 IU/ml) for 6 h. As shown in Figure 11, the proportion of apoptotic cells (Q2 + Q3) in the control, histone, and histone + LPS groups was 26.88 ± 1.44, 43.67 ± 1.88, and 58.50 ± 3.47%, respectively. Heparin remarkably alleviated cell apoptosis induced by histone, and the proportion of apoptotic cells (Q2 + Q3) was 16.63 ± 4.72 and 19.60 ± 3.47% in the histone + heparin group and the histone + LPS + heparin group, respectively.

### Heparin Can Regulate Apoptotic Proteins in HK-2 Cells Treated With Histone

**Figure 12:**
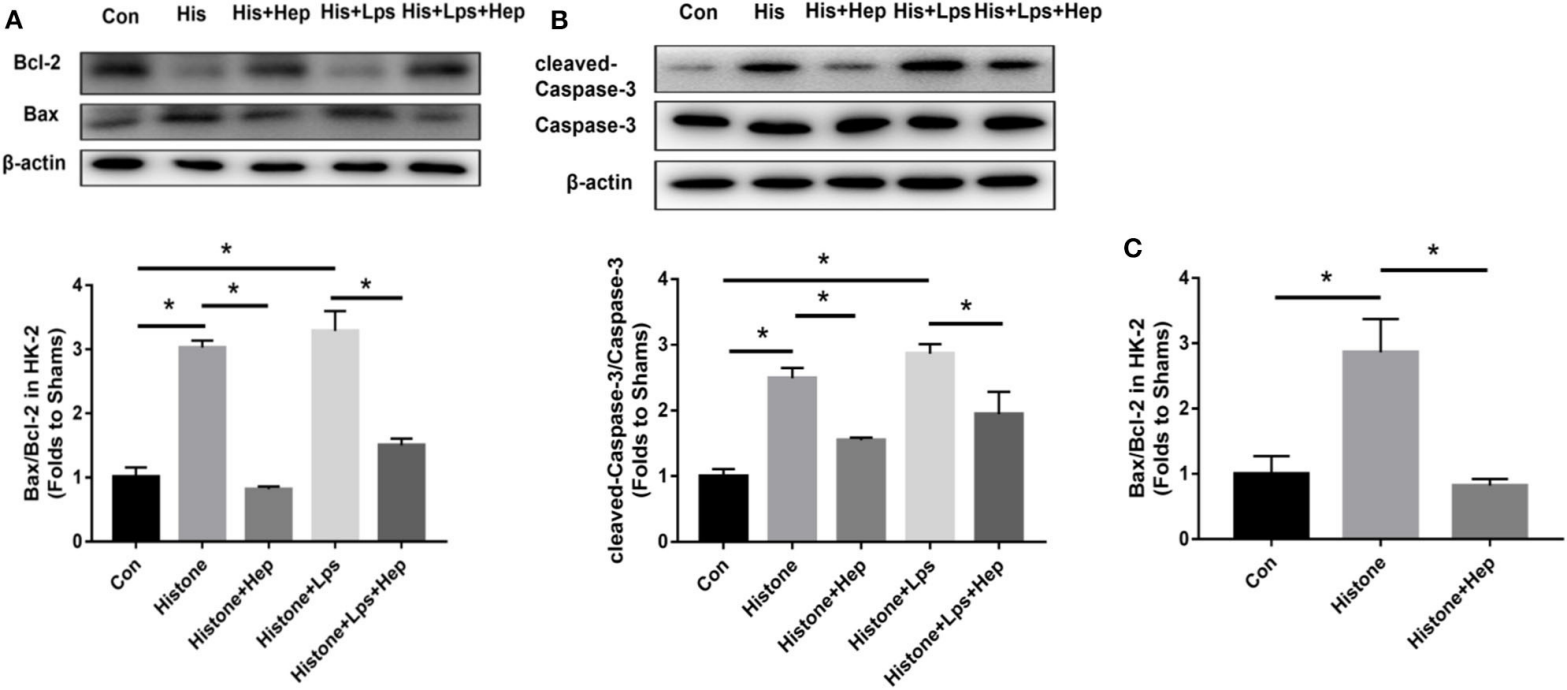
Heparin decreases the expression levels of apoptosis-mediated proteins induced by histone in HK-2 cells. **(A,B)** The protein expression of Bax/Bcl-2 and of cleaved Caspase-3/Caspase-3 in HK-2 were analyzed by western blotting and quantified by densitometry. Administration of histone enhanced the ratio of cleaved Caspase-3/Caspase-3 and the ratio of Bax/Bcl-2 in the kidney. Heparin could decrease the ratio of cleaved Caspase-3/Caspase-3 and the ratio of Bax/Bcl-2 in comparison with the histone group. **(C)** The mRNA expression of Bax/Bcl-2 was analyzed by SYBR Green qPCR. The administration of histone increased the ratio of Bax/Bcl-2 in the kidney. Heparin could decrease the ratio of Bax/Bcl-2 in comparison with the histone group. Data are presented as mean ± SD, *n* = 3 per group of the representative data from three independent experiments; *P < 0.05.

As shown in Figure 12, the ratio of cleaved Caspase-3/Caspase-3 and Bax/Bcl-2 expression was increased in the cells of the histone group, and heparin remarkably reduced the levels of apoptotic proteins. In parallel with the data obtained from western blotting analysis, our PCR results also showed that, although the ratio of Bax/Bcl-2 gene expression was dramatically increased in the cells of the histone group, the ratio decreased in samples from the heparin intervention group. The *in vitro* ratios of cleaved Caspase-3/Caspase-3 and Bax/Bcl-2 protein expression were increased in the cells of the LPS (10 μg/ml) + histone-treated group, and heparin dramatically lowered the ratios (Figure 12).

### ROS Production Is Increased During Apoptosis Development of HK-2 Cells Treated With Histone

**Figure 13:**
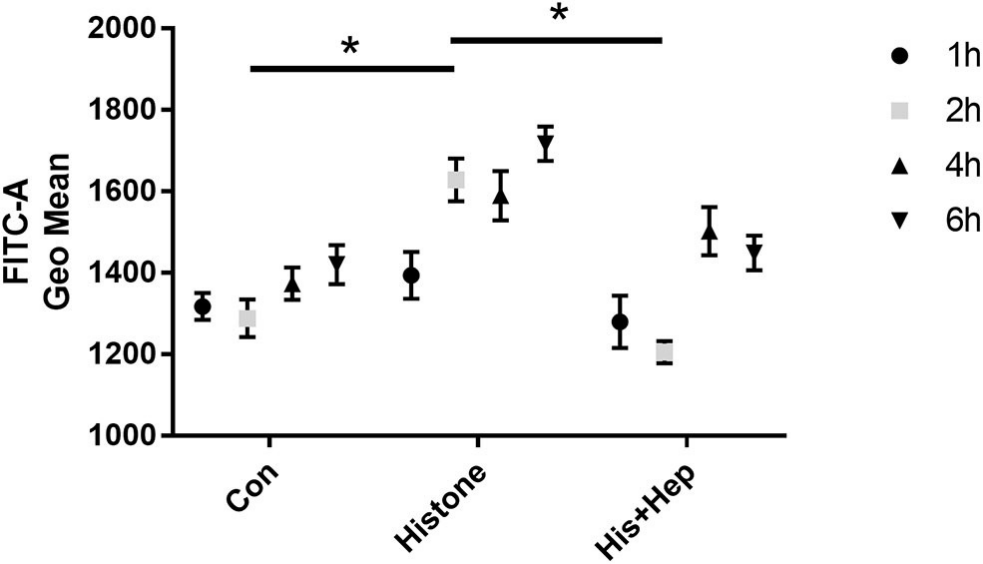
Heparin decreases the production of ROS in HK-2 cells treated with histone. In the histone + heparin group, histone and heparin were administered to the HK-2 cell line simultaneously. The cells were collected from each group at various time points (1, 2, 4, and 6 h). Histone enhanced the level of ROS production in 2 h of treatment, and heparin could decrease its level. Data are presented as mean ± SD, *n* = 5 per group of the representative data from three independent experiments; *P < 0.05.

As shown in Figure 13, we assessed the production of ROS in HK-2 cells receiving different treatments after 1, 2, 4, and 6 h. After a 2-h histone (40 U) treatment, ROS production was significantly higher in treated cells than that in the control group, and ROS production was significantly lower in cells from the heparin intervention group compared to that in the cells from the histone-treated group. There was no statistical significance in the production of ROS among the control group, the histone-treated group, and the heparin intervention group at 1, 4, and 6 h.

## Discussion

Sepsis-3 is defined as life-threatening organ dysfunction caused by disorders of the immune response (9, 10). Hence, the alleviation of organ dysfunction is crucial to improve septic prognosis. About 40–50% of patients with sepsis have AKI (11). Although the apoptosis observed in tubular epithelial cells is considered to play an important role in contributing to SAKI, the underlying disease pathogenesis has not been fully elucidated.

Histones are the main structural proteins of eukaryotic chromatin. Consisting of five protein variants, namely, H1, H2A, H2B, H3, and H4, histones can be released into the extracellular matrix under pathological conditions (12). Since both H3 and H4 have been shown to be key components within the histone octamer complex and exhibit a high level of toxicity, H4 was selected as the representative histone for measurement of histone levels in our experiments (13). Under normal conditions, histones are involved in the composition of chromosomes and in the regulation of different physiological functions, such as gene expression, mitosis, and DNA repair (14). In pathological situations, histones can be released into the cytoplasm *via* two different pathways: (1) activated neutrophils that release neutrophil extracellular traps to defend from pathogens and (2) passive release from dead cells (15). While a small amount of histones can defend from pathogens, a larger amount of histones can induce inflammatory responses and organ dysfunction (16). It has been shown that high levels of circulating histones are major mediators of multiple organ dysfunction syndrome (17). Our previous clinical experimental studies have also shown that H4 concentration is inversely correlated with the prognosis of patients with sepsis (18, 19).

Highly conserved in eukaryotic cells (20), histone proteins are highly positively charged due to enriched lysine and arginine residues (21). Heparin is a mucopolysaccharide ester composed of D-glucosamine, L-iduronic acid, N-acetylglucosamine, and glucuronic acid, which are responsible for the high negative charge of the molecule (22). Clinical meta-analysis studies have shown that heparin can decrease the 28-day mortality rate and APACHE II score among septic patients and that the severity and the dysfunction of coagulation can be alleviated by heparin without any evident increased risk of bleeding (23, 24). Recently, heparin/low molecular weight heparin is reported to have a beneficial effect on mortality through neutralization of extracellular cytotoxic histones in COVID-19 (25). In animal studies, non-anticoagulant heparin has been shown to decrease the levels of inflammatory cytokines (26) as well as improve the survival of septic mice in a dose-dependent manner. A number of studies have found that heparin may neutralize EH, thereby alleviating the toxicity of EH (6, 27, 28). In this study, we established a murine model of SAKI with CLP and used heparin as an intervention. Our results showed that both histone injection and sepsis could decrease the 72 h-survival rate; heparin enhances the survival rate and protect kidney histology. It is indicated that histone plays a major role in the pathogenesis of SAKI, and heparin may be a potential therapy. We also observed that the sepsis group had a higher level of EH4 paralleled with a higher level of kidney injury factors and pro-inflammatory factors. However, the heparin intervention group had a remarkably lower concentration of EH4 paralleled with a lower level of kidney injury factors and pro-inflammatory factors compared with the sepsis group. Consistently, western blotting and PCR data showed that the protein and the gene expression levels of apoptotic proteins also increased in the sepsis group and decreased in the heparin intervention group. The results suggested that a positive correlation was expressed between the level of histone and the degree of kidney injury in mice of SAKI; heparin may alleviate kidney injury through neutralization of histone.

We explored next the mechanism in which histone induces kidney injury and how heparin played in this process. Histone can produce cytotoxic effects as well as directly contribute to apoptosis (29), resulting in endothelial activation and, ultimately, alteration of vascular homeostasis (30). It has been proven that apoptosis, especially the apoptosis of renal tubular epithelial cells, greatly affects the process of SAKI (31, 32). For this reason, we explored the role histone and heparin played on renal tubular epithelial cells. A flow cytometry analysis was performed to evaluate the apoptosis of renal tubular epithelial cells of mice (HK-2 cells). In the pre-study, efficient time point and effective concentration of histone, heparin, and LPS were found. At the time point of 6 h, 40 U histone, 25 IU heparin, and 10 μg/ml LPS were selected as study subjects. At 6 h after stimulation, the flow cytometry results clearly demonstrated that histones increased the apoptosis rate, and heparin could alleviate it, coinciding with western blotting and PCR results of apoptosis-related protein. The ELISA results showed that heparin could decrease the levels of histone and pro-inflammation factors in the culture supernatant of HK-2 cells pretreated with histone. We concluded that histone has a cytotoxic effect on renal tubular epithelial cells through cell apoptosis and inflammatory response; heparin could alleviate these damages through neutralization of histone. Then, we talked about the positive role to renal tubular epithelial cells under the background of SAKI. As we all know, LPS is the main component of the cell wall of Gram-negative bacilli, which can trigger the activation of an inflammatory response and the release of cell mediators. It has been used to stimulate cells to induce sepsis in many studies (33, 34). We stimulated HK-2 cells with histone and LPS to simulate the pathologic state of SAKI, and it could be seen that heparin still dramatically alleviates the apoptosis and inflammation response of HK-2 cells. It is partly indicated that heparin may become a potential therapy for SAKI patients.

Additionally, we assessed the production of ROS in HK-2 cells with different treatments of histone and heparin after 1, 2, 4, and 6 h. Our results showed that, while ROS production was significantly higher in cells after a 2-h treatment with histone, ROS production was alleviated in cells in the intervention group compared with the histone-treated group. To some extent, our results suggested that the cytotoxic effect of EH on HK-2 cells may be related to mitochondrial oxidative stress that may have been manifested through the endogenous apoptosis pathway (32). Changes in myocardial mitochondria have been suggested to be the causative factor rather than sepsis-related inflammation (35). Therefore, whether ROS production can induce or influence apoptosis requires further studies.

All in all, our data showed that histone may induce SAKI through cell apoptosis and inflammation response, and heparin could play a protective role by attenuating it.

### Limitations

There are limitations in our study. Considering that our experimental data were insufficient in analyzing the mechanism of apoptosis, further validation is required. More quantitative data and evidence of apoptosis, for example, by TUNEL stain or immunofluorescence, are also limited to show in our renal section. We just indicated that histone play a more essential role *in vitro* than LPS in SAKI when the concentration of LPS is 10 μg/ml; more effective concentrations of LPS should also be taken into consideration. The positive role of heparin on HUVEC or primary mouse kidney cells of SAKI *in vitro* should be explored in a future study. While most studies addressed the correlation between EH and sepsis based on experimental animal and cell studies, there have been only very few clinical studies. Whether histone is a therapeutic target for SAKI patients requires more validation from clinical data.

## Conclusion

Taken together, our study demonstrated that histone may contribute to the development of SAKI through the activation of the apoptotic pathway and by increasing the inflammatory response. Heparin may represent a potential treatment for SAKI due to its ability to decrease apoptosis and the inflammatory response through attenuation of histones.

## Data Availability Statement

The original contributions presented in the study are included in the article/supplementary materials, further inquiries can be directed to the corresponding author/s.

## Ethics Statement

The animal study was reviewed and approved by Institutional Animal Care and Utilization Committee of Tianjin Medical University.

## Author Contributions

GL, SS, and LW contributed to the conception of this study. ZW and LW performed the experiment. CC, HJ, and YZ contributed to analysis and manuscript preparation. ZW, LW, YG, and CC performed the data analysis and drafted manuscript. GL, SS, and XL edited, revised, and approved final version of manuscript. All authors contributed to the article and approved the submitted version.

## Conflict of Interest

The authors declare that the research was conducted in the absence of any commercial or financial relationships that could be construed as a potential conflict of interest.
